# Optical and electrical recording of neural activity evoked by graded contrast visual stimulus

**DOI:** 10.1186/1475-925X-6-28

**Published:** 2007-07-04

**Authors:** Luigi Rovati, Giorgia Salvatori, Luca Bulf, Sergio Fonda

**Affiliations:** 1 Department of Information Engineering, University of Modena and Reggio Emilia, Via Vignolese 905, I-41100 Modena, Italy; 2 Department of Biomedical Sciences, University of Modena and Reggio Emilia, Via Campi 287, I-41100 Modena, Italy

## Abstract

**Background:**

Brain activity has been investigated by several methods with different principles, notably optical ones. Each method may offer information on distinct physiological or pathological aspects of brain function. The ideal instrument to measure brain activity should include complementary techniques and integrate the resultant information. As a "low cost" approach towards this objective, we combined the well-grounded electroencephalography technique with the newer near infrared spectroscopy methods to investigate human visual function.

**Methods:**

The article describes an embedded instrumentation combining a continuous-wave near-infrared spectroscopy system and an electroencephalography system to simultaneously monitor functional hemodynamics and electrical activity. Near infrared spectroscopy (NIRS) signal depends on the light absorption spectra of haemoglobin and measures the blood volume and blood oxygenation regulation supporting the neural activity. The NIRS and visual evoked potential (VEP) are concurrently acquired during steady state visual stimulation, at 8 Hz, with a b/w "windmill" pattern, in nine human subjects. The pattern contrast is varied (1%, 10%, 100%) according to a stimulation protocol.

**Results:**

In this study, we present the measuring system; the results consist in concurrent recordings of hemodynamic changes and evoked potential responses emerging from different contrast levels of a patterned stimulus.

The concentration of [HbO2] increases and [HHb] decreases after the onset of the stimulus. Their variation shows a clear relationship with the contrast value: large contrast produce huge difference in concentration, while low contrast provokes small concentration difference. This behaviour is similar to the already known relationship between VEP response amplitude and contrast.

**Conclusion:**

The simultaneous recording and analysis of NIRS and VEP signals in humans during visual stimulation with a b/w pattern at variable contrast, demonstrates a strong linear correlation between hemodynamic changes and evoked potential amplitude. Furthermore both responses present a logarithmic profile with stimulus contrast.

## Background

Several methods to investigate brain activity have been proposed to date [1]. The most common techniques can be classified in terms of the task measured: neural activity or hemodynamics. Magnetoencephalography and electroencephalography measure neural activity directly via magnetic and electrical fields exhibiting excellent temporal resolution but poor spatial resolution [2,3]. In contrast to these methods, emission tomography, functional magnetic resonance and near-infrared spectroscopy measure hemodynamic changes associated with neural activation [4,5]. In particular, emission tomography measures cerebral metabolic rate of oxygen and cerebral blood flow, whereas functional magnetic resonance measures blood flow and blood oxygenation level dependent (BOLD) changes. Both these methods offer excellent spatial resolution but limited temporal performance. On the other hand, near-infrared spectroscopy overcomes the temporal resolution limitation of these techniques offering nonetheless intermediate spatial resolution performance.

An important feature of all these techniques is their contrast-to-noise ratio, i.e. task-induced change relative to background noise. Each method has peculiarities that emphasize certain mechanisms involved in stimulus perception or interpretation, e.g. electrical/magnetic mechanisms, mechanical/thermal mechanisms or hemodynamic mechanisms. Many variables come into play in the gamut of brain activities, and thus the integration of different techniques may represent an appropriate strategy.

Motion artefacts must be addressed for all these techniques, even if some methods, such as electroencephalography, are relatively less sensitive to motion as compared to others, e.g. the combination of electroencephalography and near infrared spectroscopy may provide a more reliable interpretation of brain activity.

Over the last decades, great improvements have been achieved in the investigation of the human brain by these techniques [1-5]. Nevertheless, it would be advantageous to simultaneously observe both neural and hemodynamic activity. The design of such instrumentation might also encourage more cross talk among scientists studying the intricate complexities of this process.

In this framework, we present a system combining a continuous-wave near infrared spectrometer (NIMO, NIROX srl, Italy) and an electroencephalography system. A single personal computer is used to record and process signals and to control the visual stimulation during the experiments. This design allows comparison between neural electrical activity (evoked potential signals) and hemodynamic response (near infrared spectroscopy) triggered by the same stimulation.

After a discussion on the basic theory, measuring principle and system configuration, we present an application of the instrument through experimental results obtained from stimulation of the human visual system with different contrast levels.

## Methods

### A. Measuring principle

Near infrared spectroscopy and electroencephalography have a complementary role in extracting spatial and temporal information on direct (electrical) or indirect (metabolic) neural activity. Near infrared spectroscopy, which measures the light absorption spectra of hemoglobin, directly measures changes in blood volume and blood oxygenation supporting neural activity [4]. Electroencephalographic technique is sensitive to variations in electrical potential in brain tissue caused by neuron depolarization and repolarization [2]. These methods reflect different aspects of the underlying physiological mechanisms, which include both vascular metabolic and electrophysiological processes mutually interacting in the visual cortex during visual stimulation.

### B. Near infrared spectroscopy

Near Infrared Spectroscopy (NIRS) is a well-known optical technique first proposed by Franz Jöbsis in 1977 for *in-vivo *investigation of tissue oxygenation [6]. NIRS is based on the transparency of tissue to light in the spectral range between 700 and 1000 nm combined with the chromophore content of tissues: mainly water, deoxyhemoglobin and oxyhemoglobin.

It has been well documented that NIRS is a useful technique for investigating cerebral hemodynamic changes in humans [4]. However, due to the relatively high scattering of the skull and white matter coefficients, it is difficult for NIR photons to penetrate the head for a depth greater than few centimetres. For this reason, NIRS is essentially limited to assessing cortical function [7,8].

The hemodynamic changes measured with NIRS are often called slow signals because they occur within seconds after brain activity begins. It has also known that NIRS has the capability to non-invasively measure neural activity [9-11]. Compared with the slow hemodynamic response, the neural signal (named "fast signal") occurs within milliseconds after stimulation. Researchers suggest that this signal originates from action potentials and the consequent swelling of the neural cells or from a brief period of anaerobic metabolism that somehow alters tissue transparency [12].

Theory describing the migration of NIR photons through tissue is widely discussed in the literature [13-16]. The most accurate model is based on the photon diffusion equation that can be solved assuming that photons in tissue perform a random walk dominated by the high value of the scattering coefficient. This assumption is reasonable in near-infrared spectral region since the transport scattering coefficient *μ*'_*s*_(*λ*) is about 50 times larger than the absorption coefficient *μ*_*a*_(*λ*) in human tissues.

In our previous paper [17], we proposed to combine this model with the knowledge of water concentration in human tissue. This approach allows the absolute variations of *μ*_*a*_(*λ*) and *μ*'_*s*_(*λ*) as well as changes in oxygenated and deoxygenated hemoglobin concentration (Δ[HbO2] and Δ[HHb], respectively) to be measured according to the following system of equations:

[Δμa(λ1)Δμa(λ2)Δμa(λ3)]≅[αHbO2(λ1)αHHb(λ1)αHbO2(λ2)αHbO2(λ3)αHHb(λ2)αHHb(λ3)][Δ[HbO2]Δ[HHb]],
 MathType@MTEF@5@5@+=feaafiart1ev1aaatCvAUfKttLearuWrP9MDH5MBPbIqV92AaeXatLxBI9gBaebbnrfifHhDYfgasaacH8akY=wiFfYdH8Gipec8Eeeu0xXdbba9frFj0=OqFfea0dXdd9vqai=hGuQ8kuc9pgc9s8qqaq=dirpe0xb9q8qiLsFr0=vr0=vr0dc8meaabaqaciaacaGaaeqabaqabeGadaaakeaadaWadaqaauaabeqaceaaaeaacqqHuoariiGacqWF8oqBdaWgaaWcbaGaemyyaegabeaakmaabmaabaGae83UdW2aaSbaaSqaaiabigdaXaqabaaakiaawIcacaGLPaaaaqaabeqaaiabfs5aejab=X7aTnaaBaaaleaacqWGHbqyaeqaaOWaaeWaaeaacqWF7oaBdaWgaaWcbaGaeGOmaidabeaaaOGaayjkaiaawMcaaaqaaiabfs5aejab=X7aTnaaBaaaleaacqWGHbqyaeqaaOWaaeWaaeaacqWF7oaBdaWgaaWcbaGaeG4mamdabeaaaOGaayjkaiaawMcaaaaaaaGaay5waiaaw2faaiabgwKianaadmaabaqbaeqabiGaaaqaaiab=f7aHnaaBaaaleaacqWGibascqWGIbGycqWGpbWtcqaIYaGmaeqaaOWaaeWaaeaacqWF7oaBdaWgaaWcbaGaeGymaedabeaaaOGaayjkaiaawMcaaaqaaiab=f7aHnaaBaaaleaacqWGibascqWGibascqWGIbGyaeqaaOWaaeWaaeaacqWF7oaBdaWgaaWcbaGaeGymaedabeaaaOGaayjkaiaawMcaaaabaeqabaGae8xSde2aaSbaaSqaaiabdIeaijabdkgaIjabd+eapjabikdaYaqabaGcdaqadaqaaiab=T7aSnaaBaaaleaacqaIYaGmaeqaaaGccaGLOaGaayzkaaaabaGae8xSde2aaSbaaSqaaiabdIeaijabdkgaIjabd+eapjabikdaYaqabaGcdaqadaqaaiab=T7aSnaaBaaaleaacqaIZaWmaeqaaaGccaGLOaGaayzkaaaaaqaabeqaaiab=f7aHnaaBaaaleaacqWGibascqWGibascqWGIbGyaeqaaOWaaeWaaeaacqWF7oaBdaWgaaWcbaGaeGOmaidabeaaaOGaayjkaiaawMcaaaqaaiab=f7aHnaaBaaaleaacqWGibascqWGibascqWGIbGyaeqaaOWaaeWaaeaacqWF7oaBdaWgaaWcbaGaeG4mamdabeaaaOGaayjkaiaawMcaaaaaaaGaay5waiaaw2faamaadmaabaqbaeqabiqaaaqaaiabfs5aenaadmaabaGaemisaGKaemOyaiMaem4ta8KaeGOmaidacaGLBbGaayzxaaaabaGaeuiLdq0aamWaaeaacqWGibascqWGibascqWGIbGyaiaawUfacaGLDbaaaaaacaGLBbGaayzxaaGaeiilaWcaaa@9B90@

where *λ*_1_, *λ*_2 _and *λ*_3 _represent the wavelengths of the photons used to explore the tissue, whereas *α*_*HbO*2 _and *α*_*HHb *_are the extinction coefficients of oxyhemoglobin and deoxyhemoglobin, respectively. The solution of this system is achieved by minimizing the least square errors.

### C. Electroencephalography

ElectroEncephaloGraphy (EEG) is a common technique for recording the scalp surface electrical signal originating from electrical activity of the underlying brain structures. It includes complex information coming from all cerebral areas, and the signal amplitude, due to various somato-sensorial stimulation, is maximal for electrodes located around the {associated/underlying} brain area. Essentially, the occipital area produces a response, as an EEG variation, for visual stimulation. Consequently, an international standardization for electrode placement has been introduced with the 10–20 System: the electrode positions O_1 _and O_2 _correspond to the left and right occipital hemisphere, respectively. A total visual field stimulation induces a similar response in both locations, which can be used to study visual functioning.

However, when attempting to extract the visual response signal from the EEG, the very low signal-to-noise ratio imposes the need to acquire several EEG recordings to obtain reliable data and to process the pooled signals by means of averaging techniques to detect and evaluate specific evoked signals. Therefore, to improve the signal-to-noise ratio, the brain response must not be corrupted by other synchronous neural activities during boxcar averaging. When the sensory system involved in stimulation is the visual system, the response obtained from EEG through averaging is defined Visual Evoked Potential (VEP). The averaging algorithm implies the need for repetitive stimulation; depending on the stimulation intensity, normally 40 to 100 stimulations are needed to recover the VEP. The stimulation intensity is normally denoted according to some parameter of the stimulus applied; in our case we chose contrast, defined as L1−L2L1+L2
 MathType@MTEF@5@5@+=feaafiart1ev1aaatCvAUfKttLearuWrP9MDH5MBPbIqV92AaeXatLxBI9gBaebbnrfifHhDYfgasaacH8akY=wiFfYdH8Gipec8Eeeu0xXdbba9frFj0=OqFfea0dXdd9vqai=hGuQ8kuc9pgc9s8qqaq=dirpe0xb9q8qiLsFr0=vr0=vr0dc8meaabaqaciaacaGaaeqabaqabeGadaaakeaadaWcaaqaaiabdYeamnaaBaaaleaacqaIXaqmaeqaaOGaeyOeI0IaemitaW0aaSbaaSqaaiabikdaYaqabaaakeaacqWGmbatdaWgaaWcbaGaeGymaedabeaakiabgUcaRiabdYeamnaaBaaaleaacqaIYaGmaeqaaaaaaaa@37A1@, where L1 and L2 are the luminance values of adjacent areas of the stimulus. The VEP is interpreted as a function of time, VEP(t); its RMS value is defined as:

VEPrms=1N∑k=1NVEPk2,
 MathType@MTEF@5@5@+=feaafiart1ev1aaatCvAUfKttLearuWrP9MDH5MBPbIqV92AaeXatLxBI9gBaebbnrfifHhDYfgasaacH8akY=wiFfYdH8Gipec8Eeeu0xXdbba9frFj0=OqFfea0dXdd9vqai=hGuQ8kuc9pgc9s8qqaq=dirpe0xb9q8qiLsFr0=vr0=vr0dc8meaabaqaciaacaGaaeqabaqabeGadaaakeaacqWGwbGvcqWGfbqrcqWGqbaudaWgaaWcbaGaemOCaiNaemyBa0Maem4Camhabeaakiabg2da9maakaaabaWaaSaaaeaacqaIXaqmaeaacqWGobGtaaWaaabCaeaacqWGwbGvcqWGfbqrcqWGqbaudaqhaaWcbaGaem4AaSgabaGaeGOmaidaaaqaaiabdUgaRjabg2da9iabigdaXaqaaiabd6eaobqdcqGHris5aaWcbeaakiabcYcaSaaa@4562@

where *VEP*_*k *_(k = 1,..., N) are the discrete values of the VEP after A/D conversion. *VEP*_*rms *_is the parameter strictly related to variation of the stimulus contrast, as we will see.

### D. System description

#### 1. Block diagram and basic functions

**Figure 1:**
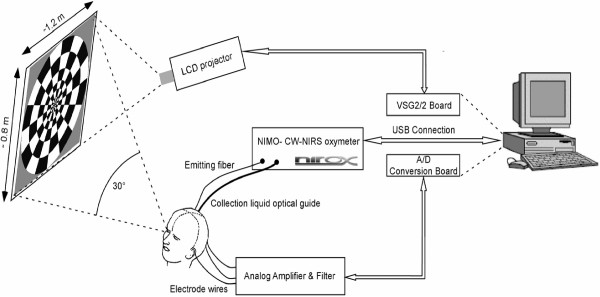
System design: (a) the optical unit is the NIMO CW-NIRS oxymeter, (b) the electrical unit includes the custom built analog amplifier and filter and the A/D converter, (c) visual stimulation and electro-optical recording interface is provided by an LCD projector, projection screen, optical head (Figure 2) and EEG-cap (Figure 3), and (d) the control unit is a personal computer running the supervisor software. The optical and electrical units controlled by the personal computer are both used to monitor the visual cortex. The same personal computer is used to control the LCD projector which generates the visual stimuli.

The block diagram of the developed measuring setup is shown in Figure 1. It consists of four main blocks: (a) optical unit, (b) electrical unit, (c) visual stimulation and electro-optical recording interface, and (d) control unit.

#### 2. Optical unit

In our previous paper [17], to overcome coupling between the scattering and absorption coefficients, we described a NIR-CWS instrument that allows quantitative assessments exploiting precise absorption measurements close to 975 nm, the absorption peak of water. Moreover, this system exploits an original detection scheme based on a time variant filter that approaches optimum shaping (in terms of signal to noise ratio) and has good rejection of photodetector offset, ambient light, and signal fluctuations due to probe movements. In this study we use the commercial release of our system produced by a spin-off company of our University: NIROX srl.

To monitor two types of blood chromophores, we employed three laser diodes emitting different wavelengths in the near-infrared spectral region. The emitted radiation from each laser diode source is collimated into a single fiber. The back-diffused light is collected by a 3 mm diameter liquid optical guide (VIS-NIR 77634, Oriel Instruments, Germany).

The receiver unit was designed to maximize the signal-to-noise ratio (SNR), reject continuous and alternate ambient light and reduce the effects of artefacts induced by optical head movement. It included a band-pass optical filter (650–1000 nm), high-sensitivity large area avalanche photodiode module (C5460-01, Hamamatsu, Japan), shaping network described in our previous paper [18], A/D converter (27.7 Hz) and USB interface to the PC.

#### 3. Electrical unit

The EEG signals were acquired using a cap with Ag/AgCl electrodes, amplified through a custom analog conditioning system (0.1–100 Hz bandpass filter at 12 dB/octave, gain of 50.000) and digitized at 250 Hz per channel, through an A/D NI card (AT-MIO16E) plugged into the PC. The software, based on the abWindows/CVI NI platform, enables control and display of the acquired EEG and processed VEP signals in real time.

#### 4. Electro-optical recording interface and visual stimulation

Great care was devoted to develop a suitable and compliant optical head. The design guidelines were focused on obtaining optimal fiber-to-skin coupling to avoid excessive pressure and motion artefacts. Moreover, since the optimal measuring position and fiber placement are not known *a priori*, we designed a variable fiber harness that allows different source-detector configurations and precise placement on the brain region of interest. The developed optical head consists of two aluminum elements: a Hollow Cube (HC) fixed on a polystyrene helmet and a mobile Fiber Holder (FH). The HC offers a matrix of 8 × 8 different horizontal and vertical locations. This geometry allows 16 different vertical sites and 16 horizontal sites to be measured over the occipital area, with a distance between two consecutive locations of 0.6 cm. This hollow cube was designed to orient the fiber harness and thus fibers orthogonal to the skin surface. The FH element can be positioned and fixed to the HC element using a pair of screws.

**Figure 2:**
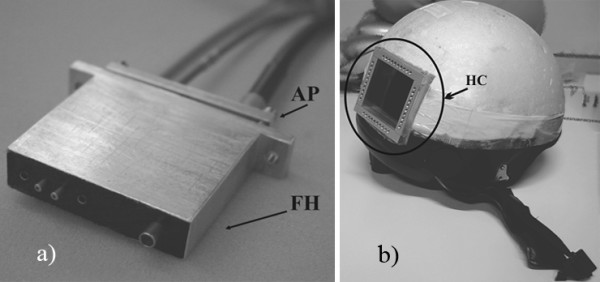
The fibre harness (FH) allows precise positioning of the illuminating fibers and collection liquid light guide a). The helmet illustrates the hollow cube (HC) with the 8 × 8 possible locations over the visual cortex region b).

The FH can hold up to four illumination multimode fibers and a collection liquid guide, as illustrated in Figure 2. Its surface is black to prevent unwanted back reflected light. To prevent discharge of the fibers from the optical head once in contact with the tissue, we inserted an o-ring for each fiber between the FH and a fixing Aluminum Plate (AP in Figure 2).

**Figure 3:**
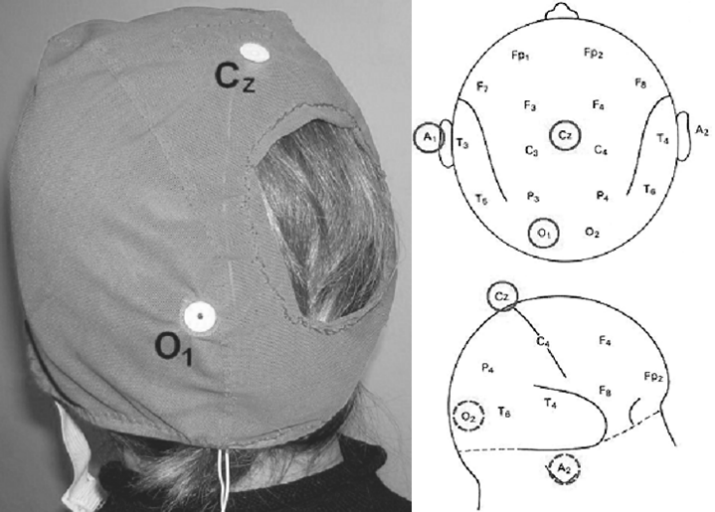
EEG-cap and the relative electrodes locations. The VEP signal is recorded from O1 and referred to Cz a), whereas A1 is the ground reference according to the standard 10–20 system positions for electrodes b).

As shown in Figure 3, a modified EEG-cap was designed with a hole in the right side to house the CW-NIRS optical head over the occipital area.

Stimuli are generated through a VSG2/2 (CRS ltd) video card driven by custom software enabling control of luminance, contrast, spatial frequency and reversal frequency.

The stimulus selected for the experiment is a windmill pattern, presenting decreasing spatial frequency content from the center to periphery, to match retinal functional organization. This pattern is projected on a large screen (1152 × 864 mm) by an LCD projector at an angle of 30 degrees to the subject's eyes. Optical stimulation consists of the reversal, at constant overall luminance, of a black and white (b/w) checkered pattern at a rate of 8 Hz.

#### 5. Control unit and signal processing

The supervisor software provides timing signals for the start and duration of the stimulation and rest periods and controls the signal acquisition of both the CW-NIRS and EEG systems. The system is composed of two modules. One module is dedicated to visual stimulation management; through a series of panels it enables the user to drive a Cambridge Instruments VSG/2 card installed on a PC to display the spatial frequency alternating at 8 Hz with appropriate intensity and contrast on a monitor. The second module runs CW-NIRS and EEG acquisitions and performs digital signal filtering.

### E. Experimental protocol

Nine subjects ranging in age from 20 to 57 years were examined. Each volunteer gave informed consent to participate in our study and was adequately informed on the protocol details.

All measurements were performed while subjects were lying in a quiet room with dimmed light; after visual adaptation, the experimental procedure was started as soon as a stable baseline for both oxyhemoglobin and deoxyhemoglobin concentration was reached.

Subjects were given different visual patterns. Because of the symmetry of the pattern stimulus in the visual field, similar VEP responses are obtained in the right and left brain hemisphere among healthy subjects [19]. Thus the EEG-cap was positioned under the polystyrene helmet and the occipital EEG channel was placed in the standard O_1 _location, whereas the CW-NIRS optical head was positioned over the standard O_2 _location. This recording layout is shown in Figure 3, considering that the calcarine sulcus varies widely in relation to cranial landmarks [20]. Therefore, the optical probe was placed vertically over the right occipital region at the level of the calcarine sulcus. The emitting fiber was positioned 1 cm to the right of the midline to avoid the sagittal sinus, and 3.5 cm from the collection liquid guide. Exploiting the different locations available in the HC fixed in the helmet, we determined that this was the best geometry to record signals associated with visual cortex activity.

All measurements were performed while subjects were lying in front of the screen. During the resting periods, a grey screen with the same total luminance of the stimulation pattern and with a central fixation cross was displayed. The subjects were asked to focus on the center of the monitor throughout the experiment.

**Figure 4:**
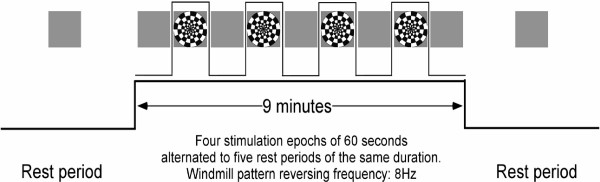
Experimental protocol adopted for visual stimulation. During each stimulation epoch, the windmill pattern is reversed eight times per second.

The experimental protocol consisted in four measurement epochs. Each epoch included a twofold presentation: a 60 second "rest" period without stimulation, followed by the inverting windmill pattern, lasting 60 seconds at a frequency of 8 Hz. Figure 4 illustrates the four measurement epochs and the corresponding visual stimulation.

The relationship between stimulus contrast and VEP amplitude is well known [21]. Our aim was to verify if a similar relationship holds between hemodynamic response and contrast parameter; so we presented each subject three stimulus-contrast levels. We assumed the maximum contrast achievable from our LCD projector to be 100%; the contrast value could be varied at 1% and 10% by the commands of the VSG/2 graphic card software.

## Results

### A. Optical recording

The optical signals acquired by the CW-NIRS were converted into concentration changes of oxygenated and deoxygenated hemoglobin (Δ[HbO2] and Δ[HHb] respectively) according to the theory described in our previous paper [17]. Data sets for each volunteer were analysed individually and expressed in micromolar concentrations (μM). The signals to be analysed were detrended to remove baseline drifts. The raw data clearly showed a marked increment of the response amplitude when the stimulus passed from the grey to windmill pattern; another relevant aspect was the feature related to heartbeat and other low frequency fluctuations (less than 1 Hz). However, no clear fast response could be observed in any subject.

To detect possible higher frequency components (starting from 8 Hz, the reversal frequency of stimulation) a non-parametric FFT algorithm was used on the [HbO2] signals. The analysis was extended to only Δ[HbO2] since previous studies show that spontaneous fast oscillations are most prominent in the [HbO2] signal [22]. We transformed two time windows of 40 seconds: the first located in the first rest period, whereas the second was chosen during stimulation at maximum contrast.

**Figure 5:**
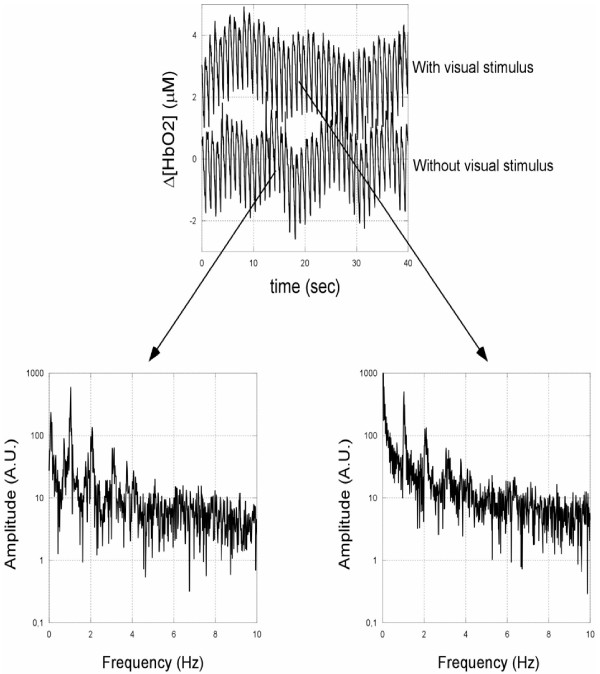
Oscillatory changes in Δ[HbO2] together with the corresponding power spectral analysis for one subject, typical of the whole group. A pronounced peak at the heart rate frequency around 1 Hz is clearly visible. Around the stimulation frequency, i.e. 8 Hz, both spectra are essentially featureless.

Figure 5 shows the oscillatory changes in [HbO2] together with the corresponding power spectral analysis for one subject, typical of the whole group.

The power spectrum signal shows relevant components in a variety of frequency bands. There is a pronounced peak at the heart rate frequency around 1 Hz (P-waves), a broad peak at the breathing rate around the range at 0.3–0.4 Hz (R-waves) and a peak in the range around 0.1 Hz (M-waves) [23].

Furthermore, harmonic components that have coherent properties with these signals can be observed. This distribution of slow hemodynamic trends indicates that light penetrates into cortical tissue [24].

Around 8 Hz both spectra were essentially featureless, thus, as reported by other researchers [24], we did not observed the fast optical response to the windmill stimulation. However, as discussed by Franceschini and Boas [25], physiological fluctuations such as arterial pulsation caused intensity changes 300–1500 times larger than those expected from the fast signal. In Figure 5, the ratio between the noise floor at 8 Hz and the arterial pulsation changes is about 1/100, thus it is reasonable that no evidence of the fast signal is present in our spectrum. Nevertheless, exploiting the high sensitivity of our instrument, this controversial aspect could be investigated by averaging several dozen trials obtained by synchronization between stimulus and epoch acquisition.

On the other hand, slow hemodynamic changes are well visible in all subjects tested.

**Figure 6:**
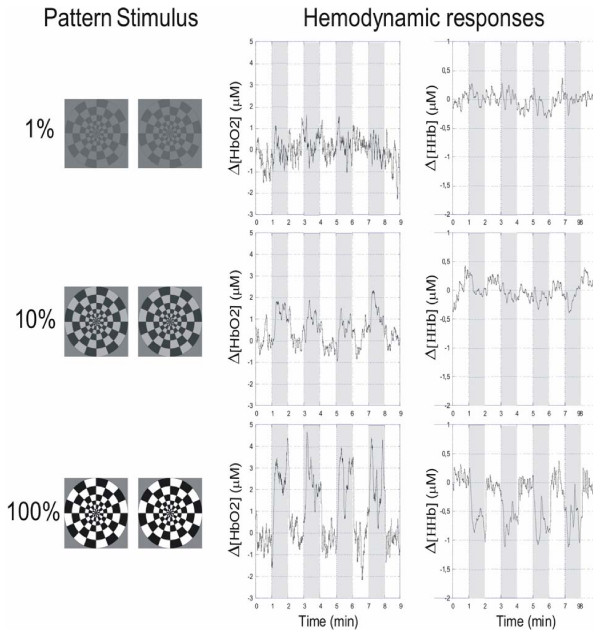
Windmill pattern stimuli and the corresponding hemodynamic responses, as acquired by the CW-NIRS system. Each line of the figure corresponds to different stimulus-contrast levels: 1%, 10% and 100%. The gray regions represent the stimulation epochs.

As an example, Figure 6 shows the signals acquired from one of the subjects as representative of typical Δ[HbO2] and Δ[HHb] responses obtained from the occipital cortex. The raw data was filtered using a moving average filter of 1.8 seconds to reduce the oscillatory changes reported in Figure 5, i.e. mainly the heart and breathing rate. As expected, the behaviour of oxygenated haemoglobin concentration reflects an increased activity of the visual cortex during the visual stimulation period. In the figure, each curve corresponds to the variation of concentration as acquired by the NIMO system using different stimulus contrasts, i.e. 1%, 10% and 100%. As shown previously in fMRI experiments [26], the hemodynamic response increases with stimulus contrast; this boost is directly dependent on the analogous bust in neural activity.

Let us consider, for example, the results obtained at maximum stimulus contrast: the recorded signal presents a reasonably stable baseline corresponding to the first 60 second non-stimulation period and variations of oxyhemoglobin concentration during stimulation. The b/w windmill pattern stimulus induced an increase in Δ[HbO2] concentration of about 3 μM. A rapid rise of the signal was observed after a short delay and then a stable level was maintained for the duration of the stimulus. [HbO2] concentration returned to baseline after about 10 seconds. Figure 6 shows that the Δ[HHb] time-course is quasi-complementary and has a very similar time scale. After an initial stable baseline, [HHb] decreased in response to stimulus in about 10 seconds. The lower concentration level remained constant up to the end of the stimulus, when the baseline condition was restored within 10 seconds.

The measuring results recorded on the pooled subjects showed a similar trend: the concentration of [HbO2] increased and [HHb] decreased after the onset of stimulation. As expected from previous studies [27], [HbO2] increased at least twofold the magnitude of the decrease in [HHb]. Hemodynamic response occurs within a few seconds after visual stimulus begins. For all subjects considered, the response reaches its maximum about 20 seconds after stimulus onset. The delay and the rise time of the signals were not apparently related to stimulus contrast. In some recordings the signals reach a plateau and decline after the stimulation period. The time course of the changes recorded is consistent with previously reported vascular responses over the occipital region [27,28].

### B. Electrical recording

To quantify neural activity during resting and stimulation conditions, we monitored the VEP signals. The alternation of black and white checkered pattern of the stimulus induced a corresponding fluctuation of the VEP signals, acquired by averaging the EEG recordings. A null response, as usual, was recorded when the subject was looking at the screen displaying a uniform grey image with the same total luminance as the patterned stimulus; this response was used as comparison with those obtained after stimulation.

**Figure 7:**
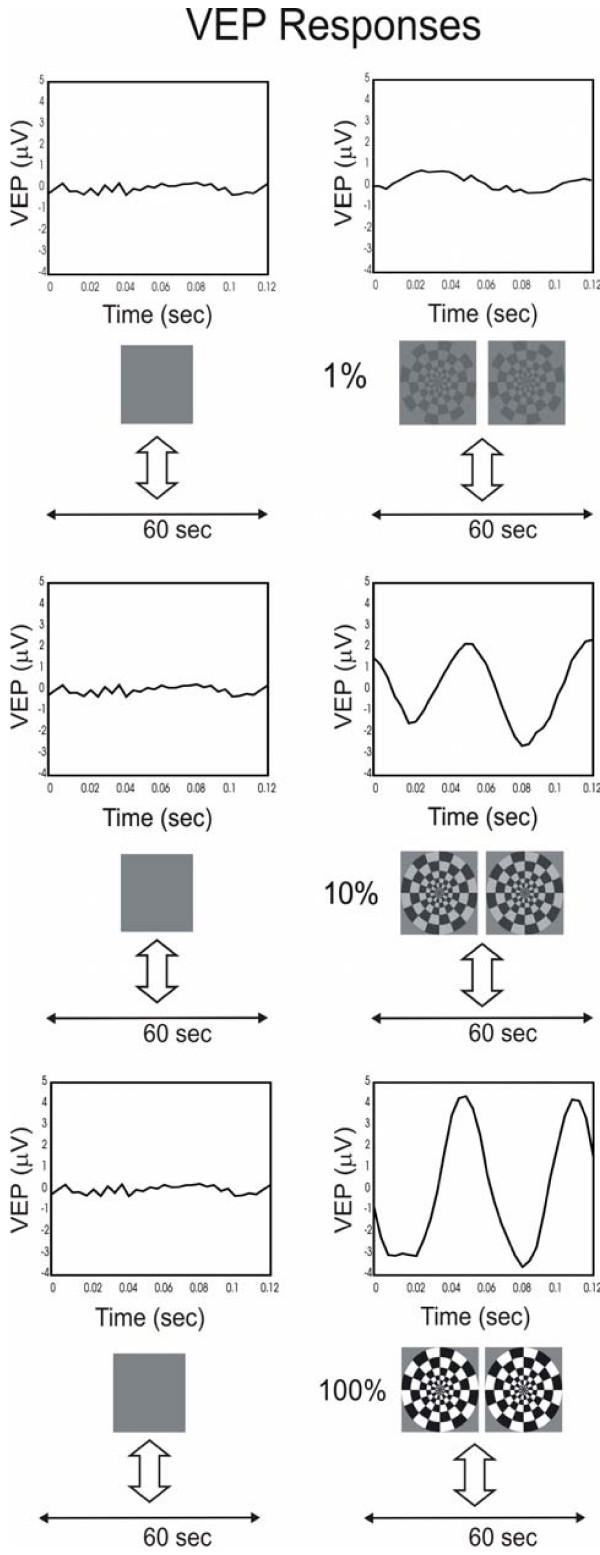
The right column shows typical VEP signals, referred to a single subject, recorded after stimulation at increasing contrast (1, 10, 100%). The left column contains VEPs recorded in a "without stimulus" condition: the subjects gaze at a uniform unpatterned screen. The large vertical arrows indicate the correspondence between the visual stimulation protocol and the recording duration, which lasts for 60 seconds. Each 60-second interval corresponds to the interval reported as a white/grey vertical strip in Figure 6.

Typical VEP signal responses are shown in Figure 7. Two different trends can be observed: during the rest condition (grey screen) there is no prominent frequency component and the VEP amplitude is roughly null. During the stimulation period the VEP signal presents a major oscillation at twice (16 Hz) the reversal trigger frequency (8 Hz): as a general rule, the retina is stimulated by pattern variations and our stimulus gives two "black and white inversions" for every triggering period. The VEP response, accordingly, presents two main oscillations, thus confirming the correlation between electrical activity and visual stimulation. The VEP amplitude increases with contrast and, despite the figure is referred to a single subject, it reflects the general property of the vision system. Similar results have been obtained in all subjects monitored. The correspondence between visual stimulation protocol and recording duration, which lasts for 60 seconds is evident in Figure 7. Each 60-second interval corresponds to the interval reported as a white/grey vertical strip in Figure 6.

As shown previously [21,29], VEP responses increased with stimulus contrast; this increase was directly attributable to the correspondingly increased neural activity.

## Discussion

The overall purpose of this paper is to combine optical and electrical recordings. Based on preliminary analyses of individual averaged waveforms, we consider the average variations in [HbO2], [HHb] and *VEP*_*rms *_that occur during stimuli and their correlation.

Filtered NIRS signals are considered to evaluate the variations of [HbO2] and [HHb] due to visual stimuli. The signal variation observed during each stimulation is calculated as the difference of the average signal during the stimulus and the average baseline recorded during the rest period. The variations observed during the four epochs were then averaged to obtain ⟨Δ[*HbO*2]⟩ and ⟨Δ[*HHb*]⟩ for each subject.

The EEG variations due to stimulus response is very small, has a poor signal-to noise ratio and cannot be directly extracted and measured from the EEG signal. This happens at every contrast value, so an averaging technique is required to record a reliable VEP. A 60-second stimulation at 8 Hz produces about 400 epochs to be averaged if an "amplitude window" is applied to the EEG signal in order to discard epochs containing excessively large amplitude variations.

All the averaged signals showed reliable trends and good signal-to-noise ratio, and they were accepted for the statistical analysis, allowing the *VEP*_*rms *_calculation for each stimulus.

**Figure 8:**
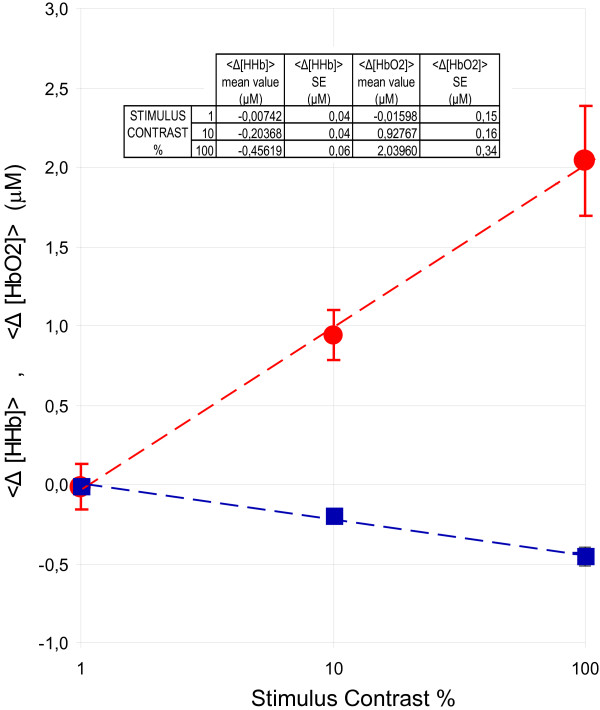
⟨Δ[HbO2]⟩ (solid red circle) and ⟨Δ[HHb]⟩ (solid blue square) averaged over observers as a function of stimulus contrast. The bars represent the standard errors (SE) evaluated over the nine subjects whereas the dashed lines represent the best logarithmic fit of the data.

The mean pooled hemodynamic variations for each stimulus contrast, averaged over observers, are shown in Figure 8. The standard errors are bounded by 0.15 μM and 0.34 μM for ⟨Δ[*HbO*2]⟩ and 0.04 μM and 0.06 μM for ⟨Δ[*HHb*]⟩. As shown in the figure, a logarithmic trend of hemodynamic response as a function of stimulus contrast is observed. Strong linear correlation (R = 0.998 and 0.997 for ⟨Δ[*HbO*2]⟩ and ⟨Δ[*HHb*]⟩, respectively) is found between ⟨Δ[*HbO*2]⟩ and ⟨Δ[*HHb*]⟩ changes and the logarithm of contrast.

**Figure 9:**
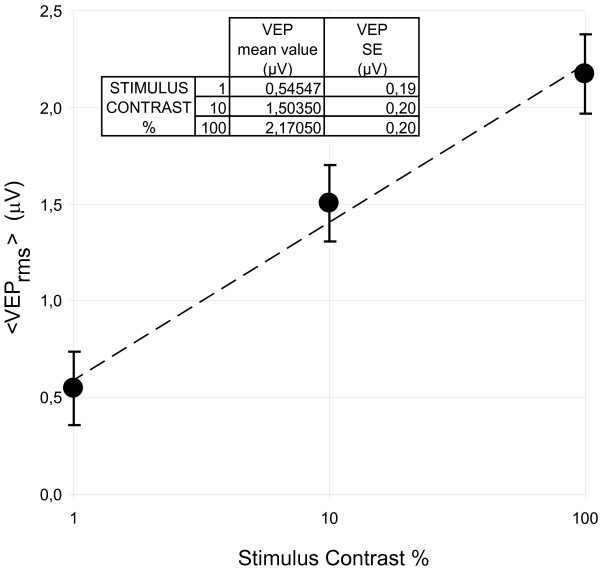
⟨ VEP_rms_⟩ (solid circle) averaged over observers as a function of stimulus contrast. The bars represent the standard errors (SE) evaluated over the nine subjects whereas the dashed line represents the best logarithmic fit of the data.

Figure 9 shows the mean electrical signal detected ⟨ *VEP*_*rms*⟩ _for each stimulus contrast, averaged over observers. The standard errors are bounded by 0.18 μV and 0.20 μV. Also in this case we observed a strong linear correlation (R = 0.994) between ⟨ *VEP*_*rms*⟩ _and the logarithm of contrast.

**Figure 10:**
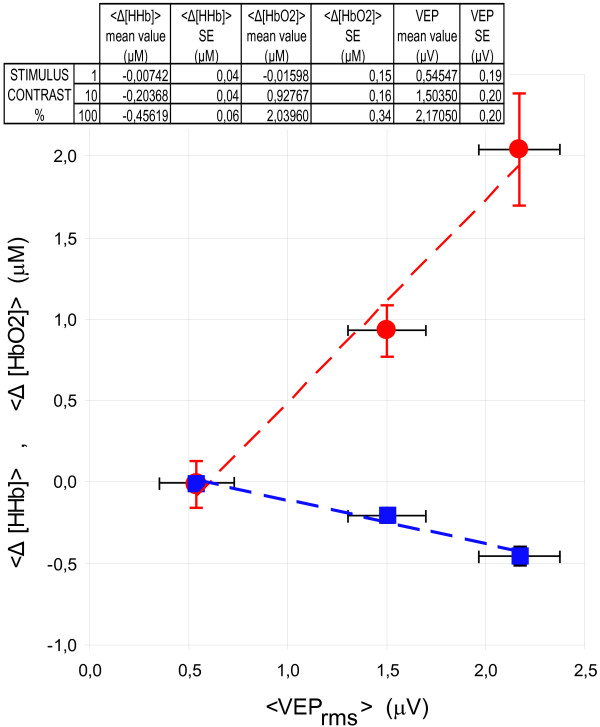
⟨Δ[HbO2]⟩ (solid red circle) and ⟨Δ[HHb]⟩ (solid blue square) as a function of ⟨ VEP_rms_⟩. The bars represent the standard errors (SE) evaluated over the nine subjects whereas the dashed lines represent the best linear fit of the data. Very small SE may be hidden by plot symbols.

There was a linear relationship between hemodynamic response and VEP response during graded visual stimulation: indeed, strong linear correlation was found between ⟨Δ[*HHb*]⟩ and ⟨ *VEP*_*rms*⟩_, as shown in Figure 10 (R = 0.988 and 0.984 for and ⟨Δ[*HHb*]⟩, respectively).

Even if the number of subjects is not sufficient to run statistical analysis, the small intrasubject standard deviation suggests that all subjects might have, in the visual cortex, roughly a similar electrical and hemodynamic response to the windmill pattern stimulation.

Neither NIRS nor electrical response is a linear function of contrast, but both increase monotonically with stimulus contrast. A "logarithmic" type of nonlinearity is evident for both the responses. Our study investigated simultaneous effects of visual contrast on hemodynamic and VEP responses. We found a strong correlation between visual log contrast and NIRS signals as well as between log visual contrast and VEP. To our knowledge, the present study is the first to address the issue of simultaneous assessment of NIRS and VEP during graded contrast visual stimulus. Nevertheless, our results are consistent with other studies, which have explored the effect of visual contrast on VEP [21,29,30]. The 'logarithmic' hemodynamic response that we observed is also in agreement with previous studies using Doppler [29] and fMRI BOLD [31].

It seems that both hemodynamics and VEP show saturation behaviour at high visual contrasts. This result is supported by animal studies of primary visual cortex neurons, which have shown that the contrast response function of spike rate responses has a sigmoidal shape. In general, neurons show an increasing response that is nonlinear at low contrasts, and a linear increase up to a certain contrast level at which the response reaches its asymptote [32-34].

Like the neural responses, fMRI BOLD signal was also found to be a nonlinear function of stimulus contrast [31]; however, a linear system analysis on the fMRI responses predicted a linear relationship between the hemodynamics and neural activity [35], as observed in our study.

## Authors' contributions

LR and GS carried out all the optical measurement setup and drafted the manuscript; LB and SF provided the stimulation and electrical recording software. All the authors contributed to refining the paper's content and structure. SF took care of revision according to peer review and all authors read and approved the final manuscript.

## References

[B1] Scroth G, Ozdoba CH, Remonda L (1995). Schweiz Rundsch Med Prax.

[B2] Gevins AS, Doyle JC, Cutillo BA, Schaffer RE, Tannehill RS, Ghannam JH, Gilcrease VA, Yeager CL (1981). Science.

[B3] Hari R, Lounasmaa OV (1989). Science.

[B4] Villringer A, Chance B (1997). Trends Neurosci.

[B5] Raichle ME, Mintun MA (2006). Brain work and brain imaging Annual Review of Neuroscience.

[B6] Jobsis FF (1977). Science.

[B7] Gratton G, Fabiani M (1998). Psychonomic Bulletin & Review.

[B8] Gratton G, Fabiani M, Elbert T, Rockstroh B (2003). Psychophysiology.

[B9] Steinbrink J, Kohl M, Obrig H, Curio G, Syre F, Thomas F, Wabnitz H, Rinneberg H, Villringer A (2000). Neuroscience Letters.

[B10] Gratton G, Fabiani M (2001). Trends Cognitive Science.

[B11] Franceschini MA, Boas DA (2004). NeuroImage.

[B12] Cannestra AF, Pouratian N, Bookheimer SY, Martin NA, Beckerand DP, Toga AW (2001). Cerebral Cortex.

[B13] Case KM, Zweifel PF (1967). Linear Transport Theory.

[B14] Davison B, Sykes JB (1957). Neutron Transport Theory.

[B15] Glasstone S, Edlund MC (1952). The Elements of Nuclear Reactor Theory.

[B16] Bianco SD, Martinelli F, Zaccanti G (2002). Phys Med Biol.

[B17] Rovati L, Bandera A, Donini M, Salvatori G, Pollonini L (2004). Rev Sci Instrum.

[B18] Rovati L (2000). IEEE Transactions on Circuits and Systems II: Analog and Digital Signal Processing.

[B19] Baseler HA, Sutter EE, Klein SA, Carney T (1994). Electroencephalography and clinical Neurophysiology.

[B20] Duncan A, Meek JH, Clemence M, Elwell CE, Tyszczuk L, Cope M, Delpy D (1995). Phys Med Biol.

[B21] Campbell FW, Maffei L (1970). J Physiol (Lond).

[B22] Obrig H, Neufang M, Wenzel R, Kohl M, Steinbrink J, Einhaupl K, Villringer A (2000). NeuroImage.

[B23] Müller T, Reinhard M, Oehm E, Hetzel A, Timmer J (2003). Med Biol Eng Comput.

[B24] Syre F, Obrig H, Steinbrink J, Kohl M, Wenzel R, Villringer A (2003). Adv Exp Med Biol.

[B25] Franceschini MA, Boas DA (2004). NeuroImage.

[B26] Heeger DJ (1999). Curr Opin Neurobiol.

[B27] Meek JH, Elwell CE, Khan MJ, Romaya J, Wyatt JS, Delpy DT, Zeki S (1995). Proc R soc Lond B Biol Sci.

[B28] Wenzel R, Obrig H, Ruben J, Villringer K, Thiel A, Bernarding J, Dirnagl U, Villringer A (1996). J Cereb Blood Flow Metab.

[B29] Zaletel M, Strucl M, Pogacnik T, Zvan B (2004). European Journal of Neuroscience.

[B30] Di Russo F, Martinez A, Sereno M, Pitzalis S, Hillyard SA (2001). Hum Brain Mapp.

[B31] Heeger DJ (1999). Curr Opin Neurobiol.

[B32] Carandini M, Heeger DJ, Movshon JA (1997). J Neurosci.

[B33] Sclar G, Maunsell JHR, Lennie P (1990). Vision Res.

[B34] Albrecht DG, Hamilton DB (1982). J Neurophysiol.

[B35] Boynton GM, Engel SA, Glover GH, Heeger J (1996). J Neurosci.

